# Withdrawal: Notch1 signaling sensitizes tumor necrosis factor-related apoptosis-inducing ligand-induced apoptosis in human hepatocellular carcinoma cells by inhibiting Akt/Hdm2-mediated p53 degradation and up-regulating p53-dependent DR5 expression

**DOI:** 10.1016/j.jbc.2021.100751

**Published:** 2021-05-13

**Authors:** Chunmei Wang, Runzi Qi, Nan Li, Zhengxin Wang, Huazhang An, Qinghua Zhang, Yizhi Yu, Xuetao Cao

VOLUME 284 (2009) PAGES 16183–16190

This article has been withdrawn by the authors. The Journal states that in Figs. 5*B* and 5*C*, a portion of the actin immunoblots were reused in the SMMC7721 panels. In Fig. 6*B*, si-p53 panel, the p53 immunoblot bands for ICN and parental lanes are duplicated. Also in Fig. 6*B*, si-p53 panel, the DR5 immunoblot bands for mock and parental lanes are duplicated. In Fig. S1, the Notch 2 and Notch 3 panels are duplicated. The authors state that they disagree with the Journal’s findings. This study was conducted nearly 15 years ago. The age of the work and the lack of availability of data storage makes it difficult for the authors to retrieve the requested raw data. The authors’ offer to replace the images in question with those from other experiments under the same conditions was declined by the Journal. The authors state that the above issues do not affect the interpretation of the data nor conclusions of this work. The authors state that the conclusions of this article have been confirmed by other independent studies. The authors have indicated that they plan to resubmit their findings elsewhere for publication.

